# A CMOS-Compatible Process for ≥3 kV GaN Power HEMTs on 6-inch Sapphire Using In Situ SiN as the Gate Dielectric

**DOI:** 10.3390/mi15081005

**Published:** 2024-08-02

**Authors:** Jie Zhang, Xiangdong Li, Jian Ji, Shuzhen You, Long Chen, Lezhi Wang, Zilan Li, Yue Hao, Jincheng Zhang

**Affiliations:** 1 Guangzhou Wide Bandgap Semiconductor Innovation Center, Guangzhou Institute of Technology, Xidian University, Guangzhou 510555, China; zjie010308@163.com (J.Z.); jijian@xidian.edu.cn (J.J.); youshuzhen@xidian.edu.cn (S.Y.); yhao@xidian.edu.cn (Y.H.); 2 Key Laboratory of Wide Bandgap Semiconductor Devices and Integrated Technology, School of Microelectronics, Xidian University, Xi’an 710071, China; 3 Guangdong Ziener Technology Co., Ltd., Guangzhou 510670, China; chenlong@zienertech.com (L.C.); wanglezhi@zienertech.com (L.W.); zilan@zienertech.com (Z.L.)

**Keywords:** GaN power HEMTs, 6-inch sapphire, CMOS-compatible process, reliability

## Abstract

The application of GaN HEMTs on silicon substrates in high-voltage environments is significantly limited due to their complex buffer layer structure and the difficulty in controlling wafer warpage. In this work, we successfully fabricated GaN power HEMTs on 6-inch sapphire substrates using a CMOS-compatible process. A 1.5 µm thin GaN buffer layer with excellent uniformity and a 20 nm in situ SiN gate dielectric ensured uniformly distributed *V*_TH_ and *R*_ON_ across the entire 6-inch wafer. The fabricated devices with an *L*_GD_ of 30 µm and *W*_G_ of 36 mm exhibited an *R*_ON_ of 18.06 Ω·mm and an off-state breakdown voltage of over 3 kV. The electrical mapping visualizes the high uniformity of *R*_ON_ and *V*_TH_ distributed across the whole 6-inch wafer, which is of great significance in promoting the applications of GaN power HEMTs for medium-voltage power electronics in the future.

## 1. Introduction

GaN HEMTs possess excellent material and device characteristics, such as high electron mobility, high critical electric field, and low switching losses. These features have led GaN to significant success in the field of power electronics, with widespread applications in power supplies, data centers, LiDAR systems, and consumer electronics [1,2,3,4,5]. So far, the three semiconductor materials Si, SiC, and GaN have achieved large-scale production and commercialization. Various silicon devices are used across different voltage ranges. SiC has also been commercialized in the medium-voltage range, and devices with voltages as high as 10 kV have been reported. However, owing to GaN’s superior physical properties that enable higher switching frequencies, GaN is destined to revolutionize the field of power electronics [6,7,8,9].

Currently, most commercial GaN HEMTs are based on large-diameter, low-cost Si substrates [3,10]. However, the complex buffer layer structure [11,12] and difficulty in controlling warpage in GaN HEMTs on silicon substrates limit their applications in medium–voltage power electronics. In recent years, substrates such as sapphire, SiC [13,14], SOI [15], and QST [16] have emerged. Among these, sapphire has been actively explored recently due to its low cost and high mechanical strength [17].

Recently, Transphorm reported the development of 1.2 kV GaN switches on sapphire, achieving over 99% efficiency for a 900:450 V hard-switched buck converter [18,19]. Subsequently, e-mode p-GaN gate HEMTs on sapphire substrates with a high breakdown voltage (*V*_BD_) of 1.4 kV were demonstrated [20]. Further, we have fabricated 1.7 kV d-mode GaN HEMTs on a 1.5 µm ultra-thin buffer [21] and 8 kV GaN HEMTs using CMOS-compatible processing [22], and reported e-mode p-GaN gate HEMTs on 6-inch sapphire [23], and d-mode GaN HEMTs on 8-inch sapphire [24], which are promising for ≥650 V power applications. Li et al. reported the 1.2 kV GaN half bridges monolithically integrated on sapphire substrates [25]. Lu et al. reported that GaN HEMTs fabricated on sapphire possess an ultrahigh breakdown voltage (*V*_BD_) exceeding 1.9 kV [26]. It should be noted that most research is based on small transistors that cannot be applied in real applications. Therefore, ≥3 kV GaN power HEMTs are strongly needed.

In this work, we present the epitaxy and fabrication of ≥3 kV GaN power HEMTs on 6-inch sapphire substrates using a CMOS-compatible process for the first time. Then, wafer-level uniformity is preliminarily assessed. Afterwards, the blocking capability is evaluated through off-state breakdown characterization, and the capacitance–voltage characteristics are probed. Finally, the threshold voltage instability of the devices is characterized.

## 2. Materials and Methods

**Figure 1:**
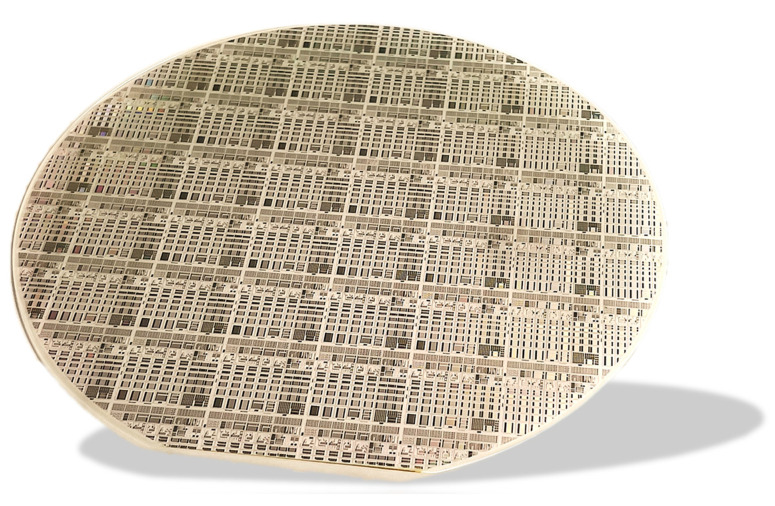
Photograph of the GaN power HEMTs on a 6-inch sapphire wafer manufactured by a CMOS-compatible process.

**Figure 2:**
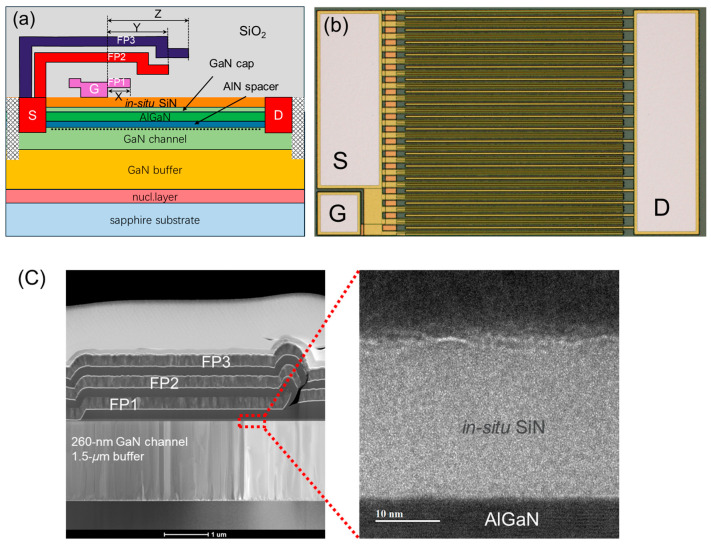
(**a**) Cross-sectional schematic of the fabricated GaN power HEMTs on 6-inch sapphire, (**b**) top view of the fabricated 36 mm GaN power HEMTs with in situ SiN as the gate dielectric, and (**c**) SEM and TEM images of the GaN power HEMTs on sapphire. The thickness of the field plate metal is approximately 300 nm.

The GaN power HEMT structure was grown on 6-inch sapphire substrates using metal–organic chemical vapor deposition (MOCVD), as shown in Figure 1, which comprises a 35 nm AlGaN nucleation layer, a 1.5 μm GaN buffer layer, a 260 nm GaN channel layer, a 1 nm AlN spacer, a 20 nm Al_0_._25_GaN barrier layer, a 5 nm GaN cap layer, and a 20 nm in situ SiN, as depicted in Figure 2a. The in situ SiN forms the gate dielectric of the transistor and also serves as the passivation layer. Figure 2c shows an SEM and TEM of the 20 nm in situ SiN and the AlGaN–in situ SiN interface.

**Figure 3:**
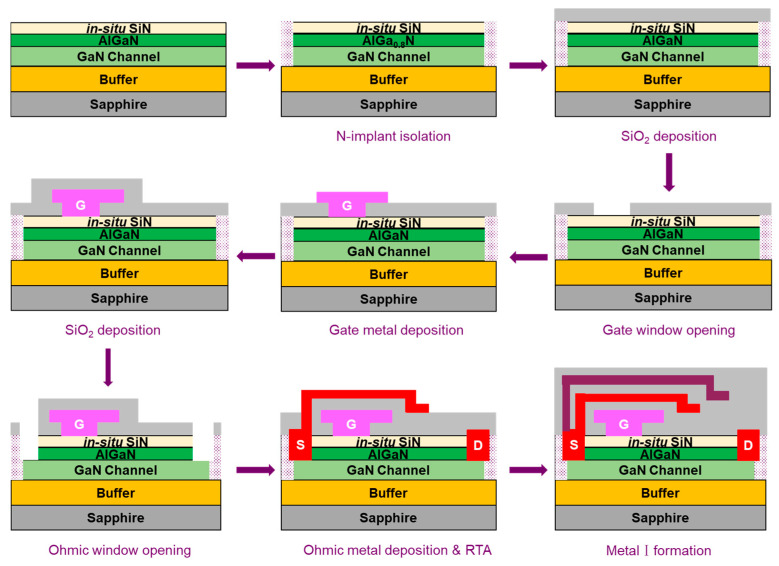
Processing flow of the GaN power HEMTs on 6-inch sapphire.

As shown in Figure 3, the device fabrication started with patterning lithography marks. Device isolation was then performed using N implantation. Next, a 150 nm SiO_2_ layer was deposited by plasma-enhanced chemical vapor deposition (PECVD), and the gate window was opened via reactive ion etching (RIE). Gate metal was deposited via physical vapor deposition (PVD), followed by gate metal patterning using inductively coupled plasma (ICP) etching. The gate metal stack comprises Ti/Al/Ti (20/250/20 nm), under which is the 20 nm in situ SiN dielectric. The gate processing was finalized by the deposition of a second SiO_2_ layer by PECVD. Next, Ohmic contact window opening was performed by RIE and ICP to remove the dielectric and AlGaN barrier, followed by Ti/Al (10/200 nm) Ohmic metal stack deposition and rapid thermal annealing at 565 °C for 90 s in the ambient of N_2_. Finally, after the deposition of a third SiO_2_ layer, Metal I of Ti/Al/Ti (20/250/20 nm) metal stack was deposited by PECVD and then patterned by ICP etching.

The fabricated power HEMTs have a gate width (*W*_G_) of 36 mm, a gate length (*L*_G_) of 4 μm, a gate–source distance (*L*_GS_) of 1.5 μm, and various gate–drain distances (*L*_GD_) ranging from 6 μm to 30 μm, as depicted in Figure 2b. The device includes three field plates, denoted as [X, Y, Z], which are formed by gate metal, Ohmic metal, and Metal I, as shown in Figure 2c. The electrical characterizations were performed using Keysight B1500A and B1505A (Keysight Technologies, one of the company’s global core sites, is located in Santa Rosa, CA, USA).

## 3. Results

**Figure 4:**
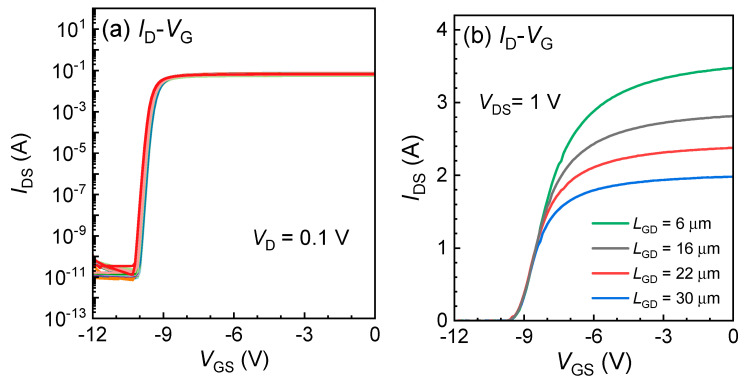
(**a**) The logarithmic and (**b**) linear transfer characteristics of the GaN power HEMTs with an *L*_GD_ of 6/16/22/30 μm on 6-inch sapphire.

Figure 4a displays the typical transfer characteristics of the GaN power HEMTs with an *L*_GD_ of 6/16/22/30 μm, on which we can see the *V*_TH_ is concentrated around −9.5 V under the criterion of *I*_DS_ = 0.1 mA/mm, attributed to the uniformly deposited in situ SiN under the gate. It can also be observed that the curves exhibit almost no hysteresis. The SS value is 104 mV/decade, and the mobility is 1561 cm^2^/V·s.

**Figure 5:**
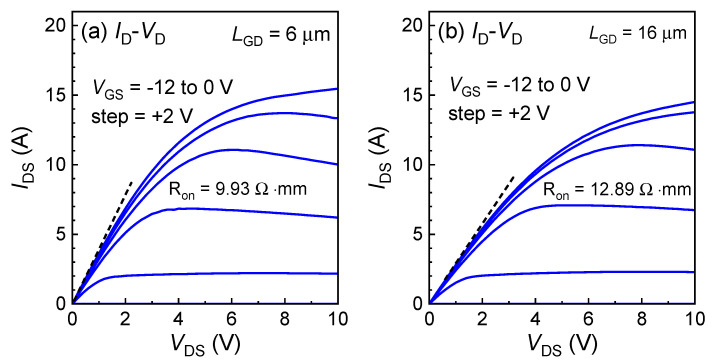

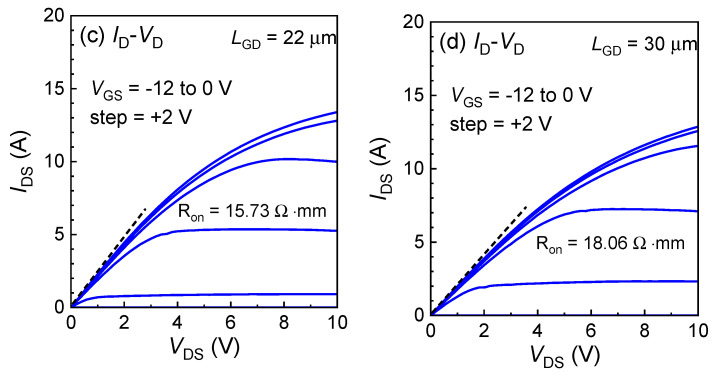
Output characteristics of the GaN power HEMTs with an *L*_GD_ of 6/16/22/30 μm on 6-inch sapphire.

Figure 5 displays the typical output characteristics of the fabricated GaN power HEMTs with various *L*_GD_ ranging from 6 to 30 μm. At *V*_GS_ = 0 V, the on-state saturation current is 15.4 A with an *L*_GD_ of 6 μm, as shown in Figure 5a. Figure 5b,c respectively show the on-state saturation current and *R*_ON_ for *L*_GD_ of 16 µm and 22 µm. The device exhibits an on-state saturation drain current of 355 mA/mm and an on-state resistance of 18.06 Ω·mm with an *L*_GD_ of 30 μm, as shown in Figure 5d.

**Figure 6:**
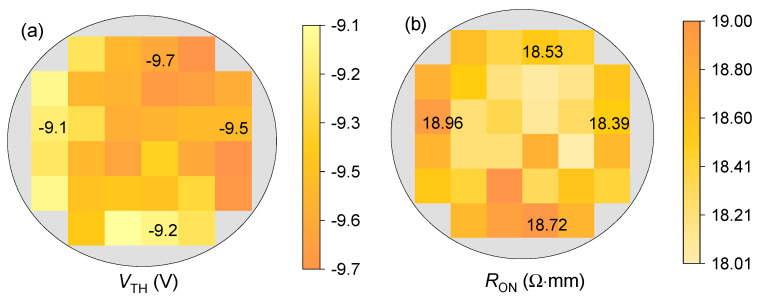
Electrical mapping of (**a**) *V*_TH_ and (**b**) *R*_ON_ of the fabricated GaN power HEMTs with an *L*_GD_ of 30 μm across the 6-inch sapphire wafer.

The electrical mapping of *V*_TH_ and *R*_ON_ was summarized to verify the uniformity of the 6-inch wafer. As shown in Figure 6, the *V*_TH_ values are concentrated in the range of −10 to −9 V, and the *R*_ON_ values are concentrated in the range of 18 to 19 Ω·mm for the GaN power HEMTs with an *L*_GD_ of 30 μm. The relatively high uniformity can be attributed to the uniform in situ SiN and the precise etch-stop technique used during gate window opening.

**Figure 7:**
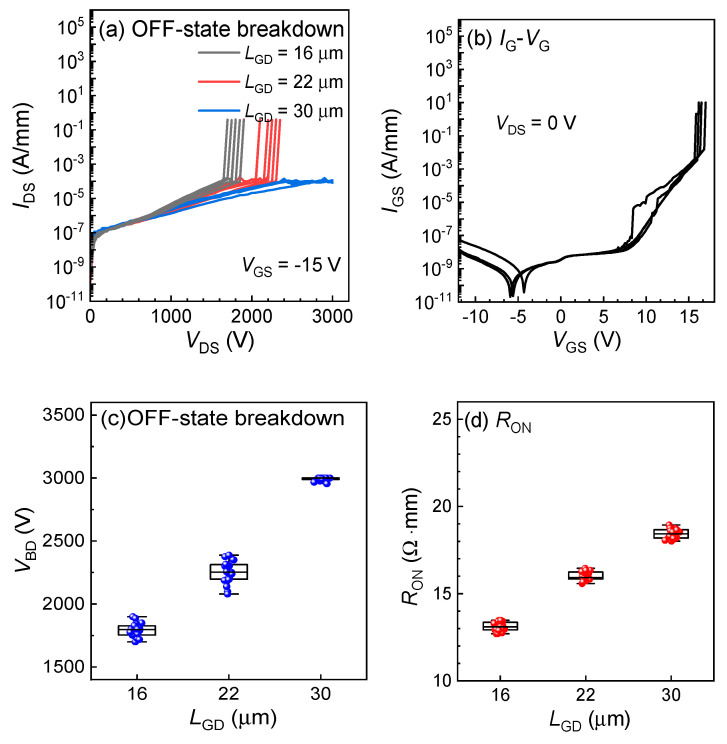
(**a**) OFF-state breakdown characteristics with various *L*_GD_ and (**b**) forward *I*_G_-*V*_G_ measurement of the GaN power HEMTs on 6-inch sapphire. The statistical distribution of *V*_BD_ (**c**) and *R*_ON_ (**d**) versus *L*_GD_.

Next, the OFF-state breakdown characteristics of the fabricated GaN power HEMTs with various *L*_GD_ ranging from 16 to 30 μm were probed. As shown in Figure 7a, generally, GaN power HEMTs exhibit excellent blocking capability, with *V*_BD_ reaching 3 kV for an *L*_GD_ of 30 μm. The boxplots of OFF-state breakdown and *R*_ON_ are presented in Figure 7c,d, respectively. Devices with different *L*_GD_ values exhibit high breakdown voltages and excellent uniformity, which highlights the potential of the GaN power HEMTs on sapphire for future medium- to high-voltage applications. As shown in Figure 7b, the gate forward breakdown voltage (*V*_GS_) reaches 16 V, owing to the high-quality and highly uniform in situ SiN. The successful fabrication of ≥3 kV GaN power HEMTs is of great significance for driving the revolution in power electronics.

**Figure 8:**
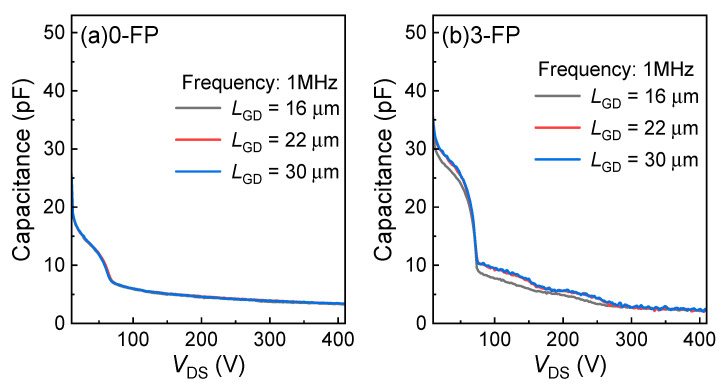
The *C*_GD_ characteristics of (**a**) the 0-FP GaN HEMTs and (**b**) the 3-FP GaN HEMTs with various structures. The measurement frequency is 1 MHz.

The capacitance–voltage characteristics (*C*_GD_) of GaN power HEMTs with different field plate structures in the OFF state were evaluated, as plotted in Figure 8. It can be seen from Figure 8a that the 0-FP HEMTs exhibit lower capacitance values, and the capacitance values remain consistent for different *L*_GD_. As shown in Figure 8b, the capacitance values for different *L*_GD_ exhibit significant differences. Moreover, the capacitances are strongly modulated by the field plate structure. Three field plate structures, [2.25 3.75 6.75], [2.25 4.75 8.75], and [2.25 4.75 8.75], correspond to *L*_GD_ values of 16, 22, and 30 μm, respectively.

Bias Temperature Instability (BTI) is a crucial reliability issue, as it generates interface traps and oxide charges that degrade the performance of HEMTs, which could trigger malfunctions in the power system during operation.

**Figure 9:**
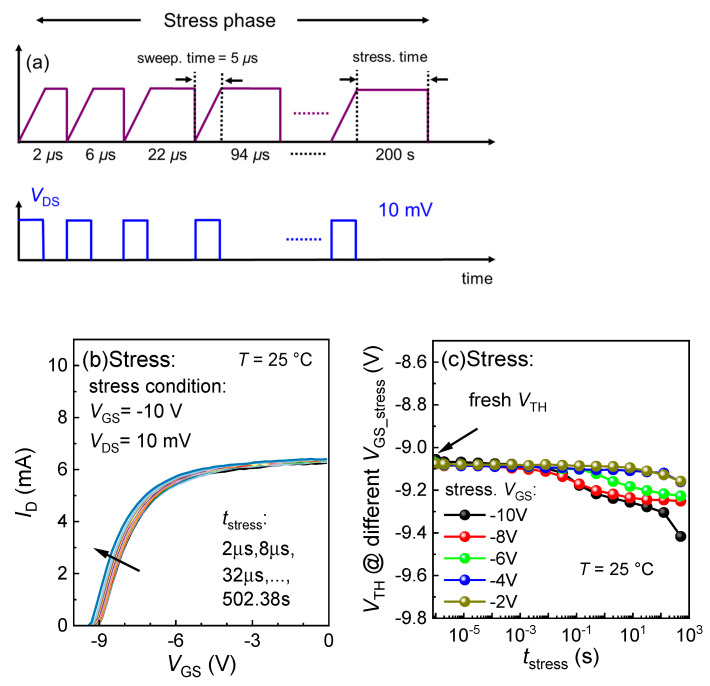
(**a**) Sketches of BTI measurement sequences using fast sweeping over 5 μs in this work, (**b**) *I*_D_-*V*_G_ curves during the stressing phase by stressing *V*_GS_ of −10 V, and (**c**) *V*_TH_ evolution under different stress *V*_GS_ from −10 to −2 V during the stressing phase at 25 °C. The fast *V*_GS_ sweeps from −10 to 0 V in 5 μs. *V*_TH_ is extracted at *I*_DS_ = 0.1 mA/mm.

In this work, BTI measurements were performed to examine the *V*_TH_ stability under gate stress using fast sweeping characterization with a Keysight B1530A WGFMU (Waveform Generator/Fast Measurement Unit). During the measurements, as shown in Figure 9a, the stress was periodically interrupted to measure the *I*_D_-*V*_G_ curves by sweeping the *V*_GS_ from −10 to 0 V within 5 μs. For negative gate stress, *V*_TH_ decreases with stressing time and the magnitude of stress voltage, as shown in Figure 9b. Figure 9c summarizes the BTI performance of the GaN power HEMTs under various *V*_GS_, and the *V*_TH_ shift was kept to as low as −0.36057 V corresponding to a *V*_GS_ of −10 V, thanks to the high-quality in situ SiN.

**Figure 10:**
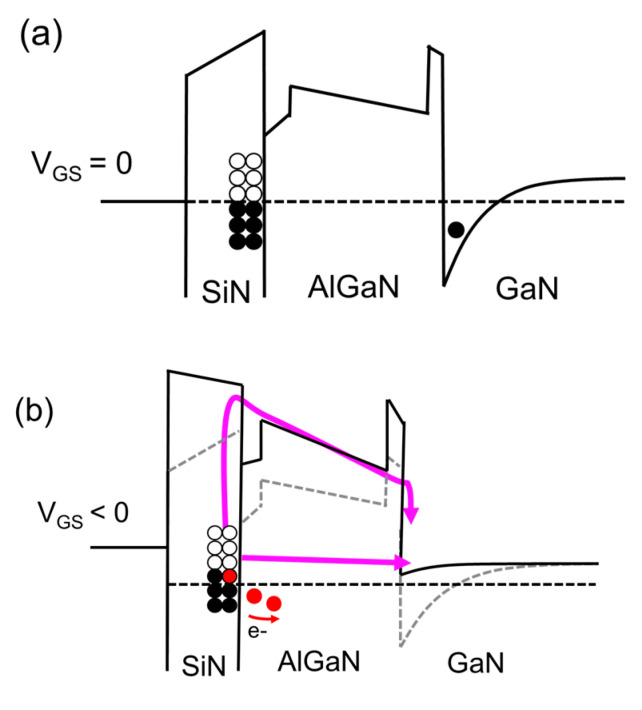
Schematic band diagram of the metal/in situ SiN//GaN/AlGaN/AlN/GaN gate stack under (**a**) *V*_GS_ = 0 V and (**b**) negative gate bias stress.

Figure 10 shows the schematic band diagram of the metal/in situ SiN/GaN/AlGaN/AlN/GaN gate stack under negative gate bias stress. The conduction band and the occupation of traps are shown in Figure 10a under thermal equilibrium, where all electrons below the Fermi level are filled. Open and filled circles indicate empty and filled traps, respectively. During BTI stress, part of the border traps in the SiN should be able to discharge, either by tunneling through the AlGaN barrier or via trap-assisted conduction, inducing a negative *V*_TH_ shift [27].

Table 1 shows a comparison of our GaN HEMTs on 6-inch sapphire substrates with other reported works. The devices in this work show excellent performance in terms of *I*_ON_/*I*_OFF_, SS, *I*_DS_, *R*_ON,SP_, *R*_ON_, *V*_BD_, and *V*_BD_/*L*_GD_. These results indicate their potential for high-power applications.

## 4. Conclusions

The GaN power HEMTs on 6-inch sapphire with an OFF-state breakdown voltage exceeding 3 kV have been successfully fabricated using a CMOS-compatible process in our pilot line. The 1.5 μm thin buffer and in situ SiN together guarantee high uniformity across the whole wafer. A preliminary evaluation of the device’s gate threshold voltage instability was conducted. The fabricated power HEMTs exhibit a low *R*_ON_, a low OFF state leakage current, and a high breakdown voltage, verifying the high quality and high uniformity of the 6-inch wafer. The remarkably simple epitaxy process and device structures, combined with the use of large-scale and low-cost sapphire substrates, can facilitate the application of GaN in the medium-voltage market in the future.

## Figures and Tables

**Table 1:** Comparison of GaN HEMTs.

Ref.	*I*_ON_/*I*_OFF_	SS (mV/Decade)	*I_DS_* (mA/mm)	*I_DS_* (A)	*R*_ON,SP_ (mΩ·cm^2^)	*R*_ON_ (Ω·mm)	*V*_BD_ (V)	*V*_BD_/*L*_GD_ (V/μm)
[20]	~10^8^	-	~350	-	6.73	17.7	1412	52.29
[26]	-	-	-	-	-	-	1925	-
[28]	10^10^	80	600	8	2.9	-	785	82.63
This work	10^10^	104	355	15.4	-	18.06	>3000	>100

## Data Availability

The data that support the findings of this study are available from the corresponding authors upon reasonable request.

## References

[1] Jones E.A., Wang F.F., Costinett D. (2016). Review of Commercial GaN Power Devices and GaN-Based Converter Design Challenges. IEEE J. Emerg. Sel. Top. Power Electron..

[2] Amano H., Baines Y., Beam E., Borga M., Bouchet T., Chalker P.R., Charles M., Chen K.J., Chowdhury N., Chu R. (2018). The 2018 GaN power electronics roadmap. J. Phys. D Appl. Phys..

[3] Chen K.J., Haberlen O., Lidow A., lin Tsai C., Ueda T., Uemoto Y., Wu Y. (2017). GaN-on-Si power technology: Devices and applications. IEEE Trans. Electron. Devices.

[4] Hoo Teo K., Zhang Y., Chowdhury N., Rakheja S., Ma R., Xie Q., Palacios T. (2021). Emerging GaN technologies for power, RF, digital, and quantum computing applications: Recent advances and prospects. J. Appl. Phys..

[5] Islam N., Mohamed M.F.P., Khan M.F.A.J., Falina S., Kawarada H., Syamsul M. (2022). Reliability, applications and challenges of GaN HEMT technology for modern power devices: A review. Crystals.

[6] Flack T.J., Pushpakaran B.N., Bayne S.B. (2016). GaN Technology for Power Electronic Applications: A Review. J. Electron. Mater..

[7] Wang J., Zhao T., Li J., Huang A.Q., Callanan R., Husna F., Agarwal A. (2008). Characterization, modeling, and application of 10-kV SiC MOSFET. IEEE Trans. Electron. Devices.

[8] Huang X., Liu Z., Lee F.C., Li Q. (2015). Characterization and Enhancement of High-Voltage Cascode GaN Devices. IEEE Trans. Electron. Devices.

[9] Stoffels S., Zhao M., Venegas R., Kandaswamy P., You S., Novak T., Decoutere S. The physical mechanism of dispersion caused by AlGaN/GaN buffers on Si and optimization for low dispersion. Proceedings of the 2015 IEEE International Electron Devices Meeting.

[10] Ishida M., Ueda T., Tanaka T., Ueda D. (2013). GaN on Si technologies for power switching devices. IEEE Trans. Electron. Devices.

[11] Selvaraj S.L., Suzue T., Egawa T. (2009). Breakdown enhancement of AlGaN/GaN HEMTs on 4-in silicon by improving the GaN quality on thick buffer layers. IEEE Electron. Device Lett..

[12] Able A., Wegscheider W., Engl K., Zweck J. (2005). Growth of crack-free GaN on Si(1 1 1) with graded AlGaN buffer layers. J. Cryst. Growth.

[13] Musolino M., Xu X., Wang H., Rengarajan V., Zwieback I., Ruland G., Messina A. (2021). Paving the way toward the world’s first 200 mm SiC pilot line. Mater. Sci. Semicond. Process..

[14] Hult B., Thorsell M., Chen J.T., Rorsman N. (2022). High Voltage and Low Leakage GaN-on-SiC MISHEMTs on a “Buffer-Free” Heterostructure. IEEE Electron. Device Lett..

[15] Jiang Q., Liu C., Lu Y., Chen K.J. High-voltage enhancement/Depletion-mode AlGaN/GaN HEMTs on modified SOI substrates. Proceedings of the 2013 25th International Symposium on Power Semiconductor Devices & IC’s (ISPSD).

[16] Li X., Geens K., Guo W., You S., Zhao M., Fahle D., Decoutere S. (2019). Demonstration of GaN integrated half-bridge with on-chip drivers on 200- mm engineered substrates. IEEE Electron. Device Lett..

[17] Integrations P. (2023). InnoSwitch3-CE Family Datasheet. https://www.powerint.cn/sites/default/files/documents/innoswitch3-ce_family_datasheet.pdf.

[18] KYOCERA (2022). Single-Crystal Sapphire Datasheet. https://global.kyocera.com/prdct/fc/product/pdf/s_c_sapphire.pdf.

[19] Gupta G., Kanamura M., Swenson B., Neufeld C., Hosoda T., Parikh P., Mishra U. 1200V GaN Switches on Sapphire: A low-cost, high-performance platform for EV and industrial applications. Proceedings of the 2022 International Electron Devices Meeting (IEDM).

[20] Cui J., Wu Y., Yang J., Yu J., Li T., Yang X., Wei J. Method to Study Dynamic Depletion Behaviors in High-Voltage (BV = 1.4 kV) p-GaN Gate HEMT on Sapphire Substrate. Proceedings of the 2023 35th International Symposium on Power Semiconductor Devices and ICs (ISPSD).

[21] Li X., Wang J., Zhang J., Han Z., You S., Chen L., Hao Y. (2023). 1700 V High-performance GaN HEMTs on 6-inch Sapphire with 1.5 μm Thin Buffer. IEEE Electron. Device Lett..

[22] Li X., Zhang J., Ji J., Cheng Z., Wang J., Chen L., Zhang J. (2024). Demonstration of >8-kV GaN HEMTs with CMOS-compatible manufacturing on 6-in sapphire substrates for medium-voltage applications. IEEE Trans. Electron. Devices.

[23] Han Z., Li X., Ji J., Chen L., Wang L., Cheng Z., Zhang J. (2024). p-GaN Gate HEMTs on 6-inch Sapphire by CMOS-Compatible Process: A Promising Game Changer for Power Electronics. IEEE Electron. Device Lett..

[24] Wang J., Li X., Chen L., Liu T., Han Z., You S., Zhang J. (2024). Report of GaN HEMTs on 8-in Sapphire. IEEE Trans. Electron. Devices.

[25] Li S., Ma Y., Lu W., Li M., Wang L., Zhang Z., Sun W. 1200 V E-mode GaN monolithic integration platform on sapphire with ultra-thin buffer technology. Proceedings of the 2023 International Electron Devices Meeting (IEDM).

[26] Lu W., Li S., Liu S., Ma Y., Zhang L., Wei J., Zhu T. Superior Performances of Dynamic On-State Resistance in 1.9 kV GaN-on-Sapphire HEMT. Proceedings of the 2023 IEEE International Conference on Integrated Circuits, Technologies and Applications.

[27] Guo A., del Alamo J.A. (2017). Unified mechanism for positive-and negative-bias temperature instability in GaN MOSFETs. IEEE Trans. Electron. Devices.

[28] Van Hove M., Boulay S., Bahl S.R., Stoffels S., Kang X., Wellekens D., Decoutere S. (2012). CMOS process-compatible high-power low-leakage AlGaN/GaN MISHEMT on silicon. IEEE Electron Device Lett..

